# Long-Term Functional Outcomes of Posttraumatic Complex Extensor Tendon Reconstruction in Hand and Forearm Using Composite Anterolateral Thigh Free Flaps: A Case Series and Literature Review

**DOI:** 10.1055/a-2844-9708

**Published:** 2026-05-29

**Authors:** Hui Yuan Lam, Wan Azman Wan Sulaiman, Arman Zaharil Mat Saad, Ahmad Sukari Halim

**Affiliations:** 1 Hospital Universiti Sains Malaysia, Jalan Raja Perempuan Zainab II, Kubang Kerian, Kota Bharu, Kelantan, Malaysia; 2 Department of Plastic and Reconstructive Surgery, Hospital Kuala Lumpur, Jalan Pahang, Kuala Lumpur, Wilayah Persekutuan Kuala Lumpur; 3 Reconstructive Sciences Unit, School of Medical Sciences, Universiti Sains Malaysia, Kubang Kerian, Kota Bharu, Kelantan, Malaysia; 4 MSU Medical Centre, Management and Science University, No. 4 Persiaran Olahraga, Shah Alam, Selangor Darul Ehsan, Malaysia

**Keywords:** extensor tendon, hand reconstruction, composite flap, Single-stage reconstruction, tendon reconstruction

## Abstract

Complex dorsal hand and forearm injuries with extensor tendon and soft tissue loss are challenging to manage. Single-stage reconstruction using composite anterolateral thigh (ALT) fasciocutaneous free flaps with vascularized fascia lata may reduce the need for multiple surgeries and accelerate functional recovery, but clinical evidence is limited. We conducted a retrospective case series of nine patients with complex extensor tendon and soft tissue defects who underwent single-stage reconstruction with composite ALT flaps between 2009 and 2018. Six patients were available for long-term follow-up (average 2–3 years). Data included demographics, injury characteristics, operative details, complications, and functional outcomes assessed using Miller's criteria. One donor site hematoma resolved without sequelae. Functional assessment revealed that two patients achieved excellent and poor outcomes in wrist and finger extension, respectively, while one patient demonstrated good and fair results . Poor outcomes were associated with missed follow-up, prolonged immobilization, and concomitant skeletal injuries. All patients returned to work and reported satisfaction with their functional status. This approach offers durable soft tissue coverage, restores tendon continuity, and allows early rehabilitation with minimal donor site morbidity.

## Introduction

The dorsum of the hand is covered by thin, non-glabrous skin and a loose areolar layer that facilitates the gliding of extensor tendons. Because of their superficial position, these tendons are highly vulnerable to degloving injuries and abrasions, most often caused by industrial accidents or motor vehicle trauma. Such injuries are frequently complicated by heavy contamination, uncertain tissue viability, and considerable morbidity. Functional restoration of the hand and forearm in these cases requires reliable soft tissue coverage, restoration of extensor tendon function, and stabilization of underlying bone.

Traditionally, management has relied on multistage procedures involving local or distant flaps or skin grafts, bone stabilization, and subsequent tendon grafting or transfer. 1 However, these approaches are associated with prolonged recovery, delayed rehabilitation, higher risks of tendon adhesions and retraction, extended hospital stays, and delayed return to work.

The advent of microsurgery has revolutionized the management of complex hand injuries, allowing single-stage reconstruction with composite free flaps or free tissue transfers incorporating vascularized tendon grafts. The primary objective is to achieve durable soft tissue coverage while maintaining tendon excursion and sufficient pull-through strength. 2 The anterolateral thigh (ALT) flap is particularly versatile and can be harvested as a chimeric or compound flap with the tensor fascia lata and adjacent muscle, enabling simultaneous tendon and soft tissue reconstruction. The vascularized fascia lata supplied by the prefascial and subfascial plexus provides a lubricated gliding surface, promotes healing, and lowers the risk of infection. 3 This study evaluates the long-term functional outcomes of composite ALT flap reconstruction for complex dorsal hand involving the extensor mechanism.

## Case

### Methods

This retrospective study was conducted in accordance with the STROBE guidelines and the Declaration of Helsinki, and was approved by the Institutional Review Board (USM/JEPeM/KK/23080619). Records of nine patients with complex hand and forearm injuries involving multiple extensor tendon defects between 2009 and 2018 were reviewed. The average follow-up period was 2 to 3 years. Data collected included patient demographics, mechanism of injury, extent of tendon involvement, operative details, complications, and secondary procedures. Informed consent was obtained for the use of clinical images.

Of the nine patients, six were available for long-term follow-up and underwent outpatient clinical and photographic assessment, while three were lost during follow-up.

Inclusion criteria included patients with complex dorsal hand or forearm injuries involving soft tissue and extensor tendon loss, who underwent single-stage reconstruction with a composite (ALT) fasciocutaneous free flap incorporating a vascularized fascia lata tendon graft between 2009 and 2018, and who had at least 2 years of documented functional outcome follow-up. Exclusion criteria were multistage reconstructions, alternative flap techniques, use of non-vascularized grafts, closed tendon injuries of non-traumatic origin, incomplete medical records, or loss to follow-up before adequate postoperative evaluation.

### Surgical Techniques

All patients first underwent meticulous wound debridement and bony stabilization with external fixation. Definitive soft tissue and tendon reconstruction with a composite ALT flap was performed after adequate wound bed preparation (typically 2–3 weeks postinjury). The composite ALT flap consisted of a free fasciocutaneous flap incorporating vascularized fascia lata, supplied by the descending branch of the lateral circumflex femoral artery (DLCFA). Vascular anastomosis was performed using end-to-side DLCFA to the radial artery and end-to-end anastomosis of the venae comitantes to the cephalic vein.

The vascularized fascia lata was divided into strips and interposed between the proximal and distal extensor tendon stumps ( Fig. 6 ). The skin paddle of the ALT flap was used to resurface the dorsal hand defect. Donor sites were closed with split-thickness skin grafts, typically 2 to 3 weeks after flap stabilization.

**Fig. 1:**
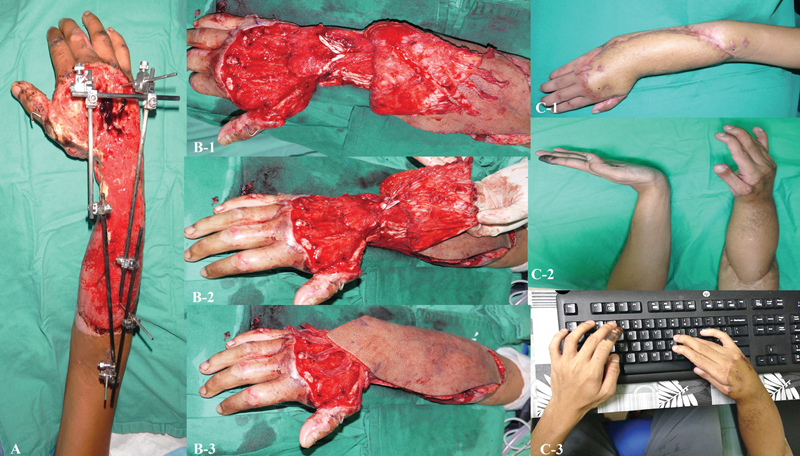
Crush injury of the forearm and hand with extensor tendon and bony loss. ( **A** ) Initial presentation showing extensive loss of multiple extensor tendons with open fractures of the right ulna and radius. ( **B** ) After 2 weeks of wound bed preparation, healthy granulation tissue was evident, indicating readiness for reconstructive surgery. ( **B-1** ) Vascularized fascia lata graft bridging the gap between proximal and distal tendon stumps. The distal end was split longitudinally into four strands and anchored to the distal tendon remnants. ( **B-2** ) The proximal fascia lata portion was similarly divided into four strands and secured to the proximal tendon stumps to restore tendon continuity. ( **B-3** ) Composite flap inset to cover the defect over the dorsum of the hand and forearm. ( **C-1, C-2** ) At 1-year follow-up, the flap showed appropriate thickness for the dorsal contour, although malunion of the forearm fractures resulted in wrist deformity. ( **C-3** ) At 9 years postreconstruction, the patient demonstrated sustained functional adaptation with compensatory movements that enabled professional work as a graphic designer.

**Fig. 2:**
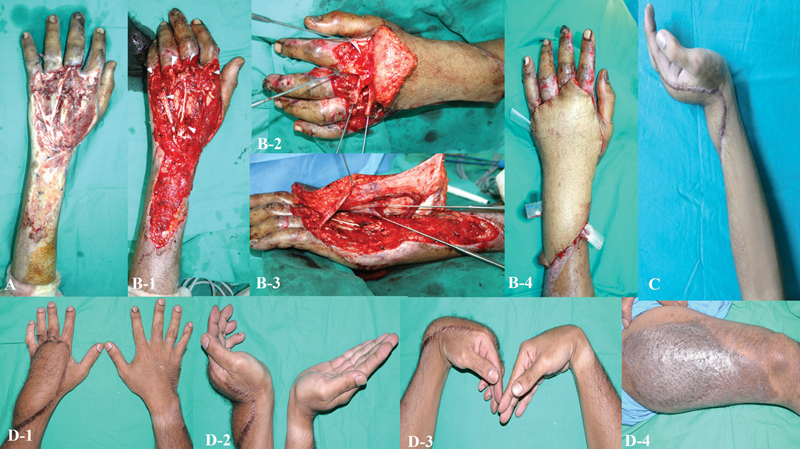
Degloving injury of the hand with tendon loss reconstructed using a composite anterolateral thigh flap. ( **A** ) Initial presentation showing an unhealthy, contaminated wound bed. **(B-1)** After debridement and 3 weeks of wound bed preparation, residual tendon stumps (white arrowheads) were identified. ( **B-2** ) Vascularized fascia lata graft split longitudinally into four strands. ( **B-3** ) Distal strands anchored to tendon stumps with 3–0 polypropylene sutures, and proximal ends secured to recipient tendons under physiologic tension. ( **B-4** ) Skin paddle contoured to match dorsal hand thickness, providing minimal bulk and optimal tendon gliding. ( **C** ) One year postoperatively, showing good wrist extension but with limited little finger extension. ( **D1–D3** ) At 14 years postreconstruction, sustained functional outcomes were observed with preserved grip strength, near-normal skin texture, and comparable flap thickness to the contralateral hand. ( **D-4** ) Donor site with complete healing and no functional deficit.

**Fig. 3:**
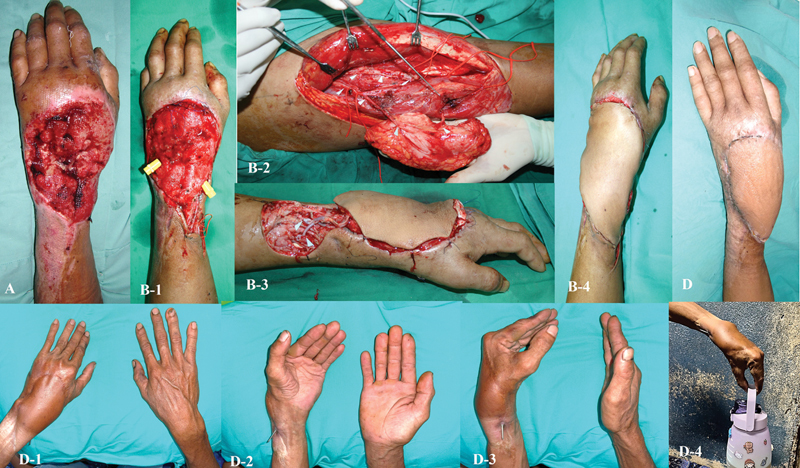
Degloving injury of the left dorsum of the hand with segmental extensor tendon loss. ( **A** ) Initial presentation showing segmental loss of extensor tendons. ( **B-1** ) Wound debridement and arthrodesis of the left wrist were performed prior to reconstruction. ( **B-2, B-3** ) Composite ALT flap inset. ( **B-4** ) Postoperative wound immediately after flap placement. ( **B-5** ) Two weeks postcomposite ALT flap reconstruction. ( **D-1 to D-3** ) Functional outcomes 15 years after reconstruction. Arthrodesis and K-wire fixation resulted in limited wrist extension and radial deviation. ( **D-4** ) Despite deformity, the patient can perform basic hand movements required for daily activities. ALT, anterolateral thigh.

**Fig. 4:**
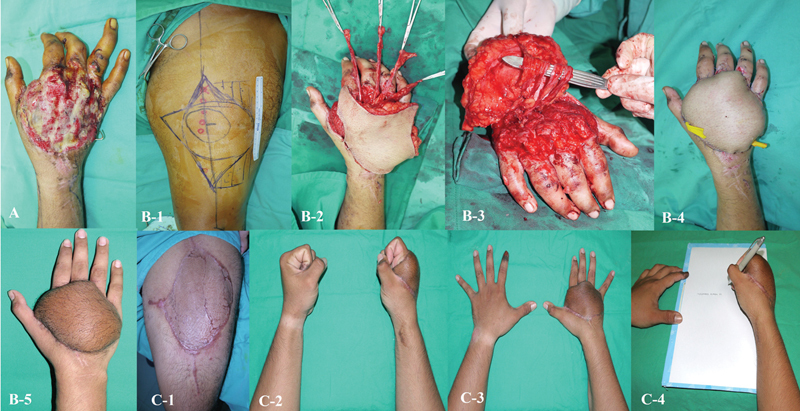
Degloving injury of the dorsum of the hand with devitalized second to fifth extensor tendons. ( **A** ) Initial wound presentation. ( **B-1** ) Design of composite ALT flap with vascularized fascia lata. ( **B-2 to B-4** ) Fascia lata sleeves were created to connect the proximal and distal extensor tendon stumps. ( **B-5** ) Immediate and 1-month postoperative view. ( **C-1 to C-4** ) Eight months postreconstruction, showing healed donor site with acceptable functional and aesthetic outcomes. ALT, anterolateral thigh.

**Fig. 5:**
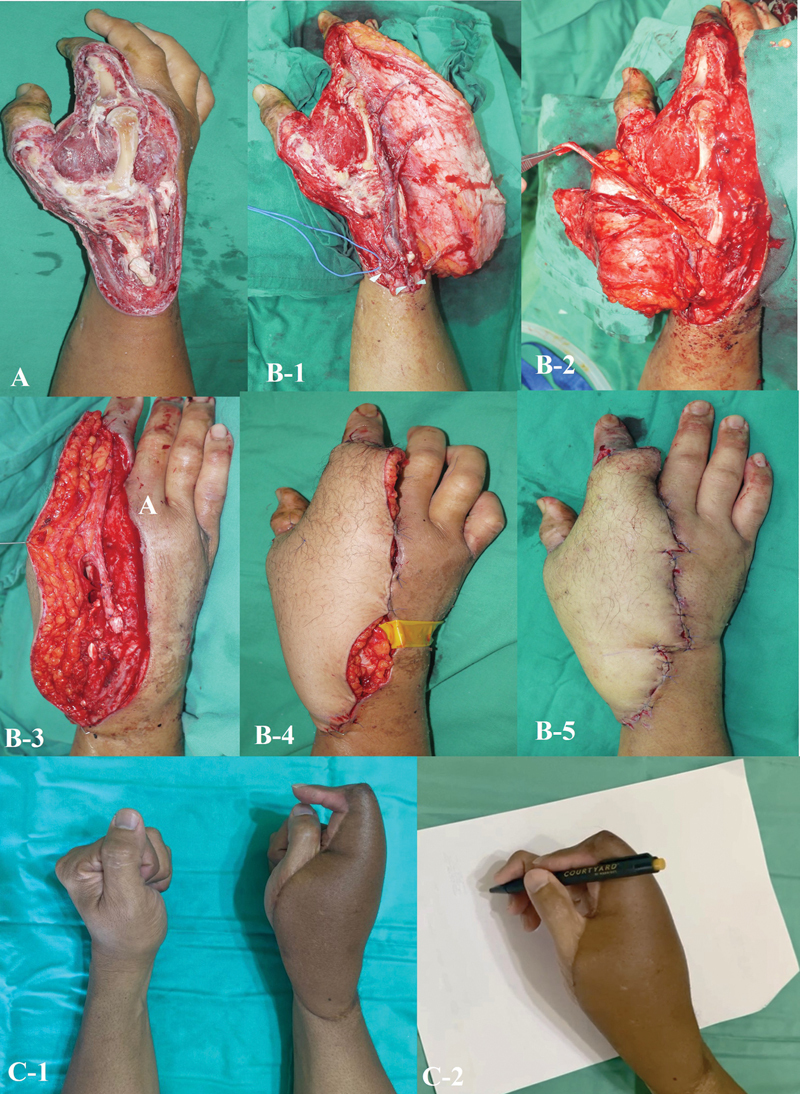
Crush injury of the right hand with metacarpophalangeal joint dislocation and open fracture of the proximal phalanx of the index finger. ( **A** ) Initial presentation showing metacarpophalangeal joint dislocation and open fracture. ( **B-1, B-2** ) Composite anterolateral thigh flap with vascularized fascia lata tendon harvested and inset; microvascular anastomosis performed to restore perfusion. ( **B-3** ) Vascularized fascia lata tendon tubularized to bridge the defects in the extensor pollicis longus and extensor carpi radialis tendons. ( **B-4** ) Postoperative view immediately after flap inset. ( **B-5** ) Secondary flap suturing was performed 10 days postoperatively to optimize tissue alignment. ( **C-1, C-2** ) Nine-year follow-up demonstrating satisfactory wrist extension but limited index finger extension due to malunion of the proximal phalanx fracture; functional impairment attributed to suboptimal bone healing rather than tendon integrity.

**Fig. 6:**
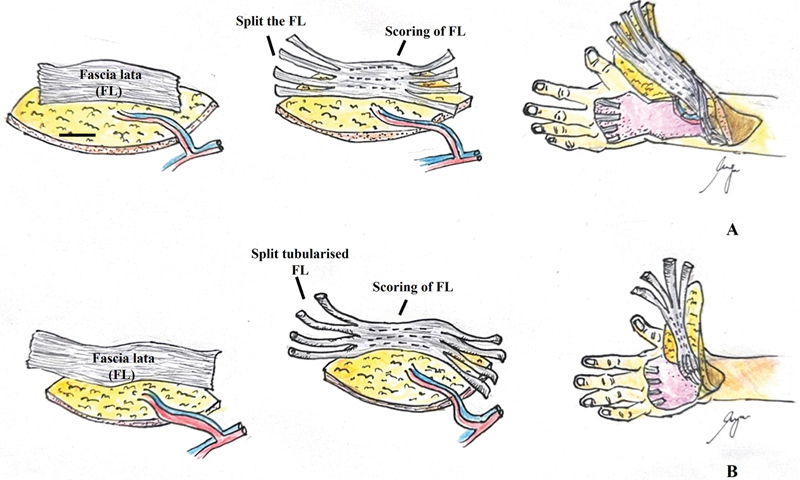
Technique for bridging tendon defects with vascularized fascia lata graft. ( **A** ) Proximal tendon gap (Zone 6 or proximal). When the defect extended proximal to Zone 6, the vascularized fascia lata graft was longitudinally split at both proximal and distal ends to span the gap. Longitudinal scoring incisions were made along the central portion of the fascia lata to maintain its attachment to the overlying skin paddle while facilitating graft flexibility. This approach ensured vascular continuity between the tendon and cutaneous components, as demonstrated in Figs. 1 to 3 . ( **B** ) Distal tendon gap (Zone 6 or distal). For defects distal to Zone 6, particularly within the dorsum of the hand, the proximal and distal ends of the fascia lata graft were tubularized and secured to the respective tendon stumps. The rationale for employing the tubing technique in distal margin defects is that an adequate length of fascia lata is available to construct the tubular structure. The central segment remained adherent to the skin paddle via controlled scoring incisions, tailored to the required tendon configuration. This method prioritized reduced tension and optimized force transmission in shorter gaps, as illustrated in Figs. 4 and 5 . Source: The illustration was created by the manuscript's first author, Lam Hui Yuan.

### Results

Six patients (four males and two females; age range 7–48 years) underwent single-stage composite ALT flap reconstruction for complex extensor tendon and soft tissue defects ( Table 1 ). The surgeries had been performed 8 to 18 years before this review. Four patients did not attend follow-up beyond 2 years, while one continued follow-up for secondary debulking. Most injuries involved extensor zones 5 to 8 and affected both superficial and deep extensor tendons. An average of four extensor tendons were reconstructed per patient, with tendon gaps ranging from 6 to 12 cm ( Table 1 ).

**Table 1:** Patient details

Patient number	Age (years)	Gender	Mechanism of injury	Tendon loss	Wound defect	Average length of tendon defect	Time for reconstructive surgery	Additional injuries	Secondary surgery
1. ( Fig. 1 )	23	Male	MVA	IF + MF + RF + LF	28 × 15 cm	12 cm	22 days	Open fracture of the (right) first to fourth carpal bones + open fracture base of first to fourth metacarpal bone + open fracture of the right ulna and radius	Capsulotomy of the second and fifth metacarpal joints
2. ( Fig. 2 )	30	Male	MVA	IF + MF + RF + LF	21 × 8 cm	10 cm	20 days	Open fracture of the proximal phalanx of the first finger Open fracture base of the first and fifth metacarpal bones Open fracture of the right radius and ulna	Flap secondary suturing Volar plate and capsule release of MF, RF, and LF over the metacarpal joint
3. ( Fig. 3 )	17	Male	MVA	T + IF + MF + RF	10 × 14 cm	6 cm	11 days	Open fracture over the proximal third of the fourth metacarpal bone	Debulking surgery
4. ( Fig. 4 )	48	Male	MVA	IF + MF + RF + LF	15 × 10 cm	6 cm	12 days	Open fracture of the right radius and ulna	Nil
5. ( Fig. 5 )	15	male	MVA	T + IF	23 × 10 CM	9 cm	9 days	Open fracture of the right proximal phalanx of the right index finger and MCPJ dislocation	Flap secondary suturing
6.	7	Female	Extension contracture secondary to TBSA 86% Full thickness Burn injury	T + IF + MF	18.5 × 8 cm	9 cm	4 years	Flexion contracture of the right and left foot Microstomia	Nil

Abbreviations: IF, index finger; LF, little finger; MCPJ, metacarpophalangeal joint; MF, middle finger; RF, ring finger; T, thumb.

All patients were treated with composite ALT fasciocutaneous free flaps incorporating vascularized fascia lata for simultaneous soft tissue and tendon reconstruction. No acute flap complications, such as venous congestion or necrosis, were observed. One patient developed a donor site hematoma, which was managed without long-term morbidity. Functional assessment using Miller's criteria ( Table 2 ) showed two patients each with excellent, good, and poor outcomes in wrist and finger extension. Poor results were associated with missed follow-up and chronic immobilization caused by retained hardware or multiple scar revisions ( Table 3 ).

**Table 2:** Miller's criteria for assessing extensor tendon function

Results	Total extension lag (degrees)	Total flexion loss (degrees)
Excellent	0	0
Good	≤10	≤20
Fair	11–45	21–45
Poor	≥45	>45

**Table 3:** Long-term functional outcomes

Patient no.	Miller's criteria	Working status	Wrist flexion	Wrist extension	Radial deviation	Ulnar deviation	Donor site morbidity
1.	Fair	Working	45	35	60	60	Well-healed scar. Normal hip flexion and abduction.
2.	Excellent	Working	100	75	50	60	Well-healed scar. Normal hip flexion and abduction.
3.	Excellent	Working	100	60	50	50	Well-healed scar. Normal hip flexion and abduction.
4.	Poor	Working	0	0	0	0	Well-healed scar. Normal hip flexion and abduction.
5.	Good	Working	45	60	50	60	Well-healed scar. Normal hip flexion and abduction.
6.	Poor	Working	45–90	0–90	0	0	Well-healed scar. Normal hip flexion and abduction.

Spearman's rank correlation was used to assess the relationship between selected variables and Miller's criteria ( Table 4 ). The analysis showed no statistically significant correlations, likely due to the small sample size ( *p* > 0.05). However, some correlations demonstrated moderate to strong magnitudes. There were moderate positive correlations between age and both the number of tendon losses and wound area, as well as between the length of tendon loss and time to surgery. Length of tendon loss also showed a strong positive correlation with wound area ( *p* = 0.076). Regarding Miller's criteria specifically, the number of tendon losses exhibited a weak positive correlation, while time to surgery showed a moderate negative correlation. All other variables had negligible correlation magnitudes.

**Table 4:** Correlation between variables and Miller's criteria

Number	Variables	Mean (SD)	Median (IQR)	1	2	3	4	5
1	Age (years)	23.8 (14.05)	20.5 (20.00)					
2	Number of tendon losses	3.5 (0.84)	4.0 (1.25)	0.54a				
3	Wound area (cm ^2^ )	209.3 (108.26)	159.0 (131.50)	0.43 a	−0.10 a			
4	Length of tendon loss (cm)	8.7 (2.34)	9.0 (4.50)	0.09 a	c	0.77 b		
5	Time to surgery (days)	255.7 (590.02)	16.0 (371.00)	−0.14 a	0.17 a	0.03 a	0.50 a	
6	Miller's criteria	2.5 (1.38)	2.5 (3.00)	−0.03 a	0.23 a	−0.03 a	0.11 a	−0.47 a

Abbreviations: IQR, interquartile range; SD, standard deviation.

a *p* > 0.10.

b *p* > 0.05.

c Not computable.

All patients reported satisfaction with their functional outcomes and were able to return to work and recreational activities. No significant psychosocial impact or donor site morbidity was noted, as evidenced by the patients' ability to participate in sports and daily activities.

## Discussion

Limb salvage in complex hand injuries is challenging, with the primary goals of restoring bony stability, ensuring tissue perfusion, and maintaining functional tendon continuity. 4 5 Each reconstruction is unique, and may be complicated by intercalary defects, aesthetic and functional concerns, and concurrent life-threatening injuries. Tendon reconstruction, in particular, requires smooth gliding surfaces and preservation of surrounding structures to achieve favorable outcomes.

Extensor tendon injuries are broadly classified as open or closed ruptures, each with distinct etiologies and management approaches. 6 Closed ruptures commonly secondary to rheumatoid arthritis, crystal deposition disease, or distal radius fractures—may be managed conservatively or with surgical repair if functional impairment is significant. 7 8 Open injuries, including avulsions, sharp lacerations, and crush trauma, generally require surgical reconstruction to preserve muscle–tendon integrity.

**Fig. 7:**
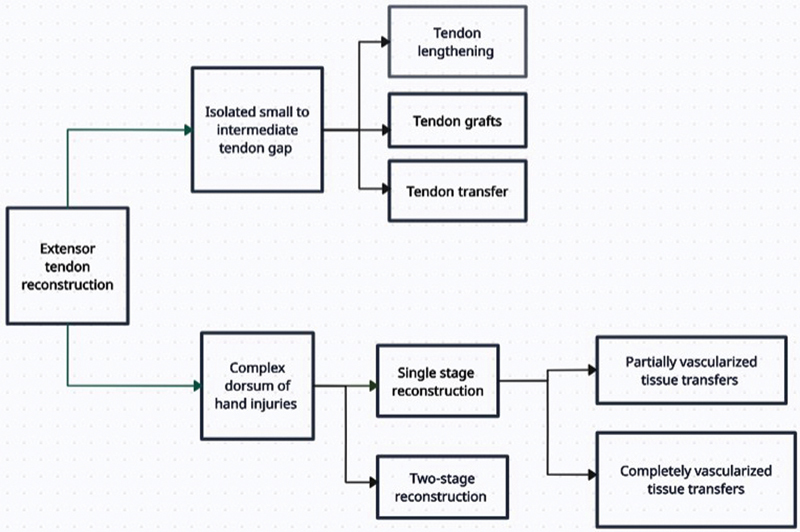
Outline of management in extensor tendon reconstruction.

Management strategies for complex dorsal hand injuries with exposed tendon, bone, and extensive soft tissue defects include multistage procedures, partially vascularized single-stage approaches, and fully vascularized single-stage reconstruction ( Fig. 7 ). The conventional multistage method involves initial soft tissue coverage, followed by tendon and, when necessary, bony reconstruction. For example, Abdulaziz et al 9 described that the use of silicone rods to create pseudosynovial sheaths allows staged tendon grafting with improved integration. However, this approach is associated with prolonged hospitalization, higher costs, extensive scarring, and extended physiotherapy.

Microsurgical composite free flaps have advanced the reconstruction of complex hand injuries by allowing single-stage procedures that shorten treatment duration and facilitate earlier return to function. 10 11 12 Scheker et al reported that single-stage composite reconstruction reduces the number of surgical interventions, expedites return to work, and minimizes morbidity, thereby enhancing societal reintegration.

Taylor and Townsend first described the composite free flap with vascularized tendon segments in 1979, outlining the criteria for an ideal composite graft: Inclusion of multiple tendons surrounded by paratenon, predictable vascular supply, thin and mobile skin, minimal donor site morbidity, and suitable vessels for microvascular anastomosis. 13 The dorsalis pedis composite free flap has produced favorable outcomes in hand reconstruction but is limited by donor site morbidity and the restricted size of its skin paddle. 10 14 15 16 The radial forearm flap with vascularized tendon is another viable option, but it is less favored because of postoperative edema and the risk of vascular compromise in injured limbs. More recently, the tripartite superficial circumflex iliac artery perforator (SCIAP) flap has been described, offering a thin skin paddle and vascularized fascia for distal thumb reconstruction. However, it is less suitable for reconstructing multiple extensor tendons or terminal tendons in distal interphalangeal joints.

The ALT fasciocutaneous flap, with its chimeric potential, is well-suited for reconstructing extensive dorsal hand defects associated with extensor tendon loss. Its narrow design permits the harvest of a thin skin paddle (as thin as 5 mm), making it ideal for dorsal hand resurfacing. 17 In addition, the vascularized fascia lata provides a durable gliding surface for tendon reconstruction, minimizing adhesions and facilitating functional recovery. 18

Functional outcomes of extensor tendon repair are commonly evaluated using Miller's criteria, which assess total extension lag and total flexion loss. Repairs performed in zones 5 to 8 generally achieve superior results compared to those from zones 1 to 4, largely because the larger proximal tendons allow core suturing with dorsal cross-stitch reinforcement, whereas the smaller distal tendons are technically more challenging to repair. In our series, all tendon injuries were in zones 5 to 8; nevertheless, functional results indicate that factors beyond anatomical location may influence recovery. The most significant predictor of reduced motion after extensor tendon repair is adhesion formation between the repaired tendon and surrounding soft tissues. Such adhesions limit tendon excursion and gliding, resulting in extension lag or flexion loss. The extent of adhesion development is shaped by several clinical factors:

- **Defect size and injury complexity** : Larger defects or lacerations accompanied by comminution cause greater soft tissue trauma and scarring, thereby increasing the risk of adhesions.
- **Associated fractures and immobilization** : In our series, three of six patients presented with concomitant metacarpal fractures. Bony injuries contribute to functional loss not only through direct metacarpal shortening—which cadaveric data suggest causes approximately 7 degrees of metacarpophalangeal (MCP) extension lag per 2 mm of shortening—but also necessitate immobilization. Immobilization, in turn, promotes fibrosis and adhesion formation. The extensor quadriga effect, described by Verdan, 19 may further exacerbate motion loss, as impaired gliding of one tendon can reduce extension across multiple digits.
- **Timing of surgery and rehabilitation initiation** : Delays in definitive repair permit progressive scarring and stiffness. Similarly, postponement of active rehabilitation prolongs immobilization and worsens functional recovery. Although early controlled mobilization protocols reduce adhesion and improve range of motion, their use is often limited by patient compliance, concomitant injuries, or surgeon preference.
- **Patient-dependent variables** : Adherence to splinting instructions, participation in hand therapy, and individual biological differences in healing significantly influence recovery. Even technically successful repairs may yield poor outcomes if rehabilitation participation is inconsistent.

Taken together, although tendon zone location predicted a relatively favorable prognosis in our cohort, several modifying factors—particularly adhesion formation driven by defect size, fracture presence, immobilization requirements, and timing of mobilization—likely account for the variability in surgical outcomes. This aligns with the findings of Newport et al, 20 who reported superior results in isolated tendon injuries compared with cases complicated by combined osseous or soft tissue trauma. 21

Our analysis was limited by incomplete radiographic correlation between metacarpal shortening and clinical lag. Nonetheless, the observed variation in patient function strongly suggests that secondary influences beyond defect length alone determine long-term results. Optimizing extensor tendon repair, therefore, requires not only meticulous surgical technique but also comprehensive management of associated injuries, minimization of immobilization whenever feasible, and early, structured rehabilitation tailored to patient compliance capacity. 21

Three of six patients also sustained metacarpal fractures, which can lead to metacarpal shortening and contribute to extension lag at the MCP joint. Cadaveric studies have demonstrated an average of 7 of MCP extension lag for every 2 mm of metacarpal shortening. The extensor quadriga effect, first described by Verdan in 1960, occurs when impaired gliding of one extensor tendon—often secondary to adhesions after fractures—reduces extension across multiple digits, as the extensor digitorum communis muscle drives four tendons simultaneously. This mechanism decreases MCP extension and may induce intrinsic muscle shortening and tightness, potentially leading to compensatory overloading at the proximal interphalangeal joint and secondary swan-neck deformity . While extensor tendon injuries in zones 5 to 8 are generally associated with more favorable outcomes owing to anatomical advantages, concomitant fractures and soft tissue complications can significantly compromise function. Comprehensive management, including careful surgical planning, patient compliance, and structured care, remains essential for optimizing recovery.

Our series demonstrates that single-stage composite ALT flap reconstruction provides satisfactory long-term functional and aesthetic outcomes in complex hand injuries. Most patients achieved good to excellent wrist extension and were able to return to gainful employment. These results are consistent with previous reports supporting the use of vascularized tendon grafts to reduce adhesion formation and enable early mobilization. While this approach restores both wrist and finger extension, the greatest functional benefit lies in reestablishing robust wrist extension. Effective wrist extension generates a tenodesis effect on finger flexion, which is critical for grip strength and overall hand function, and often represents the limiting factor in cases of extensive dorsal hand injury with combined soft tissue and tendon loss.

This study has several limitations. First, its retrospective design and small sample size limit the generalizability of the findings, and three of nine patients were lost to follow-up. Functional outcomes were evaluated using Miller's criteria; future research should incorporate patient-reported outcome measures, objective functional testing, and aesthetic evaluations to provide a more comprehensive assessment. The variability in functional results in our cohort is likely related to the complexity of crush injuries involving multiple tissue types. Compared with the uniformly high outcomes reported by Trần et al after ALT flap reconstruction for extensor tendon injuries, 18 our results were less consistent, likely due to more severe trauma, frequent concomitant skeletal injury, prolonged immobilization, and inconsistent rehabilitation compliance. 18 While other cohorts included predominantly isolated tendon and soft tissue defects managed with early mobilization, our cohort featured multiple cases complicated by metacarpal fractures necessitating extended immobilization, resulting in increased extension lag and diminished function. These factors—particularly injury severity, skeletal involvement, and timing of rehabilitation—account for the variability in outcomes and underscore the importance of comprehensive management strategies to optimize recovery after complex dorsal hand reconstruction.

### Conclusion

Single-stage reconstruction of complex dorsal hand injuries with a composite ALT fasciocutaneous free flap incorporating vascularized fascia lata is a reliable option that provides durable coverage, restores tendon continuity, and supports early rehabilitation with minimal donor site morbidity. Long-term follow-up in this series supports its role as a primary reconstructive strategy for patients requiring functional restoration and timely return to activity. Prospective studies with larger cohorts and standardized functional and patient-reported outcome measures are warranted to confirm these findings and refine patient selection.
